# A Method for Predicting Production Costs Based on Data Fusion from Multiple Sources for Industry 4.0: Trends and Applications of Machine Learning Methods

**DOI:** 10.1155/2023/6271241

**Published:** 2023-10-10

**Authors:** Masoud Soleimani, Hossein Naderian, Amir Hossein Afshinfar, Zoha Savari, Mahtab Tizhari, Seyed Reza Agha Seyed Hosseini

**Affiliations:** ^1^Department of Computer Engineering, University of Isfahan, Isfahan, Iran; ^2^Amirkabir University of Technology, Tehran, Iran; ^3^Department of Economics, Shahid Chamran University, Ahvaz, Iran; ^4^Department of Management and Economics, Science and Research Branch, Islamic Azad University, Tehran, Iran; ^5^Department of Industrial Engineering & Management Systems, Amirkabir University of Technology, Tehran, Iran; ^6^California Miramar University, School of Business, San Diego, CA, USA

## Abstract

There is a growing need for manufacturing processes that improve product quality and production rates while reducing costs. With the advent of multisensory information fusion technology, individuals can acquire a broader range of information. Several data fusion and machine learning methods have been discussed in this article within the context of the Industry 4.0 paradigm. Depending on its purpose, a prognostic method can be categorized as descriptive, predictive, or prescriptive. ANN and CNN models are applied to predicting production costs using neural networks based on multisource information fusion, and multisource information fusion theory is examined and applied to ANNs and CNNs. In this study, ANN and CNN predictions have been compared. CNN has demonstrated more remarkable skill in predicting the six cost categories than ANN. When predicting the true value of each cost category, CNN is superior to ANN. As a result, CNN's forecast error for the current month's total income is 0.0234. Because of its improved prediction accuracy and more straightforward training technique, CNN is better suited to incorporating information from several sources. Furthermore, both neural networks overestimate indirect costs, including direct material costs and item consumption prices.

## 1. Introduction

In manufacturing, the fourth industrial revolution refers to a general movement to adopt new communication systems and protocols, cyber security norms, display devices that can display multiple devices simultaneously, mobile and compact communication devices with ever-increasing computation capabilities, and artificial intelligence methods. As this international trend has grown, the Internet has expanded to permeate every facet of human life, including economics and social life [1–3]. Digital technologies have also been widely implemented within industrial manufacturing procedures and investments due to this paradigm shift. Essentially, the smart factories of tomorrow will be built on the convergence of the physical and digital worlds. Despite the growing popularity of deep learning and neural networks, there are still obstacles to combining multiple sources of data and information. Deep learning and neural networks remain challenging when combining information from multiple sources. In decision-making, Bayesian reasoning provides a rigorous method for quantifying uncertainty [4]. Bayesian inference quantifies uncertainty by combining multiple data sources and considering uncertainty related to model parameters. With multiple data sources and a Bayesian framework, Chandra and Kapoor proposed a method for transfer learning based on neural networks. They used the Markov chain Monte Carlo method to get samples from the posterior distribution in a multisource Bayesian transfer learning framework. Despite the ambiguous experimental results, the framework offers a robust probability-based foundation for decision-making. Pattern recognition and artificial intelligence communities have focused on self-centered activity recognition due to its wide application to human systems, such as dietary and physical activity assessment and patient monitoring [5, 6]. The authors created a simple probability table based on a knowledge-driven multisource fusion architecture to provide frequent information regarding self-centered activities (ADLs) in everyday life. Using statistics and support vector machines based on information theory, a well-trained convolutional neural network creates a set of text labels from regular information and other sensor data. The proposed method can accurately recognize several previously challenging sedentary activities, including 15 predefined ADL categories. Compared to previous methods, this method provides better results when applied to data collected using wearable devices. This research has not yet been widely adopted, despite an average accuracy of 85.4% for 15 ADLs.

Several robotics-related research domains have recently benefited from artificial neural networks (ANNs) because of their superior spatial feature abstraction and keyframe prediction capabilities. An ANN is a connectionist model, which makes them inherently wrong at making long-term plans, thinking logically, and making multistep decisions. In their study, Zuo et al. developed an enhanced ANN (SANN) model of state calculator and result (SOAR) that combines the feature detection abilities of ANN with the long-term cognitive planning capabilities of SOAR [7]. A logical planning module is added to the classic ANN to improve its performance by imitating the cognitive operation of the human brain. The SOAR planning probability vector was merged with the original feature array of data via a data fusion module [7–12]. Experiments have shown that the suggested architecture is efficient and accurate and has excellent potential for more challenging tasks requiring quick categorization, planning, and learning. It is possible to recognize grasping sequences when multiple objects are involved and perform metaobject cooperative grabbing. However, the benefits of these applications are limited [3]. A diagnosis based on data fusion is an exciting application of the Industrial Internet of Things for the efficient use of motor monitoring data. A multimodal neural network (DRMNN) based on dynamic routing was introduced by Wang et al. to follow the concept of deep multimodal learning (MDL) [8, 9]. They proposed a strategy for dimensionality reduction and invariant feature capture using vibration and stator current signals to extract multimodal features from multisource data. The decision-making layer implements a dynamic routing method to assign appropriate weights to various modes based on the relative relevance of each mode. DRMNN is practical and durable in a motor test platform trial.

To implement robot demonstration programming, Wang et al. suggested an implicit interaction technique based on forearm sEMG (surface electromyography) and inertial multisource information fusion [10]. An M-DDPG method for modifying assembly parameters was presented based on the demonstrator's demonstrations and lessons learned to improve adaptability to diverse assembly components. To improve generalization performance and accuracy of gesture identification, they proposed an improved PCNN (1D-PCNN) based on one-dimensional convolution and pooling to extract feature inertia and EMG. Previous studies found that retailers' prior disclosure of imprecise information flow would reduce the supply chain's profitability and cost retailers' money. By mentioning the possibility of manufacturers infiltrating and confronting uneconomical or economical manufacturing, Zhao and Li expand the study on information sharing. Manufacturing costs do not have to be addressed when retailers expropriate manufacturers and share demand information with producers [11]. A further incentive may be provided by producers to retailers in order to increase the accuracy of their demand estimations.

The manufacturer infringes and experiences production diseconomy, the retailer benefits from information exchange, and the manufacturer benefits from minimal production combined with exceptional conditions. It has not been examined whether retailers gain more from the following factors when demand becomes more variable or when demand signals become more accurate [6]. There is a tendency in the educational publishing industry to create a great deal of stock for “on-demand manufacturing,” but modifying the item might lead to obsolescence problems. He et al. addressed two distinct but related problems [12]. A variety of printed items are forecasted and managed using predictive models. Demand estimates can now be more precise, and inventory obsolescence can be reduced. Also, educational publishing merchants benefit from contracts that have knowledge asymmetries.

Consequently, profit margins have not been optimized throughout the supply chain, and manufacturer profits are also low. In order to increase the profitability of the supply chain, the report recommends encouraging merchants to provide accurate data. The suggested approach was validated based on an empirical investigation of Taiwan's top education publishers. As a result of the suggested printing choice model, prediction accuracy is increased by 3.7%, and costs are reduced by 8.3%. As a result of the contract design, the manufacturer's and supply chain's profitability increases by 0.5% and 2.7%, respectively [7]. However, the initial capital expenditure is excessive [13]. Multisource information fusion and neural networks are discussed in this study. The research has contributed to the advancement of related professions. Data analysis and methodologies can provide us with a great deal of knowledge. However, the use of neural networks to predict production costs has received very little attention. The study of this field requires a thorough implementation of these algorithms.

We present two neural network-based information fusion techniques that combine neural networks with information fusion systems. In order to combine the benefits of multisource signals with neural networks, it is necessary to integrate the multisource data processing mechanism within the neural network. Using artificial neural networks (ANNs) and conclusion neural networks (CNNs), we demonstrate the superiority of multisource data fusion over single-source data fusion. For the neural network to learn, it does not require any new data; it can assemble information from multiple sources and conduct experiments as it learns.

## 2. Related Literature

By measuring the geometric characteristics of a product, the dimension meter determines whether it meets its geometric tolerance criteria (form, orientation, profile, runout, size, and location). A dimension measurement can be obtained by rigorous monitoring methods, such as complex measuring, and automated inspection methods, such as automatic measuring machines, such as CMMs and OMPs [14–16]. Manual inspection techniques can suffer from unpredictable error sources, including operator errors, causing significant measurement errors. As a result of its efficiency, versatility, and precision, coordinate metrology has become crucial for industrial dimension metrology [17, 18]. A meaningful uncertainty statement requires considerable effort because CMM measurements are sensitive to various variables, including random and systematic influences. Also, CMSs can be used to compare coordinate measurements of workpieces with measurements of calibrated master components that have the same nominal geometry [19–22]. In order to assess the uncertainty associated with coordinate's observations, a significant percentage of the systematic effects associated with the CMS must be modeled. Since comparative coordinate measurements are based on relative measurements, it is not easy to establish the traceability route associated with them.

In addition, workpieces that inherit part of their measurement uncertainty from the calibration process of the master part will do so as well. Nevertheless, this uncertainty component is often straightforward to calculate [13, 23–25]. A nonrepeatable fixturing configuration is particularly susceptible to process variations. These can include part misalignments due to rotations of geographic coordinate frames generated during the mastering and measurement modes. In addition, calibrating a part using a calibrated CMM is often necessary to produce a master component for comparator measurement. CMMs and manual measuring tools are often used in traditional component quality evaluation techniques, creating production bottlenecks and slowing down production. As process control and monitoring methods are being developed using Artificial Intelligence (AI) methods and live monitoring data, there has been a push to minimize nonvalue-adding activities such as dimensional inspections and make timely judgments. Machine learning process models have been used to map process variables to product quality criteria, such as surface roughness, as part of Business 4.0 [26].

Based on material hardness, process parameters, and force data, an artificial neural network (ANN) can predict surface roughness and tool wear in dry hard turning [27]. A single inexpensive accelerometer sensor was used to generate vibration data that could enhance surface quality monitoring in CNC turning [28]. Plaza et al. [29] developed least squares support vector machines (LS-SVMs) based on cutting settings and tool geometry parameters. Huang developed a neural-fuzzy monitoring system for end-milling operations to predict surface roughness based on process parameters and force data. According to Huang et al. [30], surface roughness is modeled by fuzzy logic and regression analysis based on machining parameters. A factorial design was used by Kovac et al. [31] to predict cutting forces and waviness based on feed per tooth, tool diameter, and radial and axial depth of cut during thin-wall component machining. A variable-parameter drilling approach was introduced by Bolar et al. [32] for multihole components made of difficult-to-cut materials. The spindle speed, feed rate, outside corner wear, thrust force, and torque were used to predict hole surface roughness using radial basis function (RBF) networks. Based on vibration and power data, Han et al. [33] proposed a machine learning-based monitoring system for milling machines and processes. Moore et al. [34] employed Bayesian networks and ANNs to predict surface roughness in high-speed milling based on workpiece geometry, material hardness, machining parameters, and cutting forces. As a result of this classification challenge, Bayesian networks proved to be easier to read and performed better than ANNs.

Correa et al. [35] presented a multisensor DP multisensor fusion decision-theoretic method that combines force, vibration, and acoustic emission (AE) inputs to detect anomalous process drifts in ultraprecision machining. In contrast to standard classification techniques such as ANNs and SVMs, their system can identify ultraprecision machining process drifts with greater accuracy. Beyca et al. [36] developed an adaptive experimental strategy for determining the ideal combination of parameters to maximize the material removal rate by using a Bayesian learning technique. The authors of Karandikar et al. [37] proposed using vision and sound to monitor the material removal rate during the grinding process. A prediction model for material removal rate monitoring was developed with the help of a light gradient boosting machine and the best feature subsets. Wang et al. [38] proposed an online tool condition monitoring system based on sensor fusion and machine learning. Even though power and sound sensors are more informative for forecasting tool conditions than displacement and AE sensor signals, they evaluated several classification algorithms using experimental data. In a simulation of the operation of a water heater, Nazir and Shao [39] outlined a principle for monitoring systems based on Bayesian networks. Under the assumption that sensors are uncorrelated, Atoui et al. [40] developed a method of sensor monitoring based on a linear state-space model that simultaneously estimates measurement noise covariance and state variable probabilities. A quadruple water tank experiment was used to evaluate the variational Bayesian inference procedure by estimating the joint posterior distribution using two different proposal distributions.

In the ramp-up phase of an MMP, Zhao et al. [41] developed a Bayesian monitoring approach based on a linear state-space model to estimate control parameters and set cause-selecting chart limits. As suggested by Tran et al., two one-sided Shewhart-type charts are proposed to account for situations in which the production run is finite. Their solution to the quality control problem was based on simulation data from the food industry. In order to detect minor to moderate shifts in the process mean, Du et al. [42] developed Bayesian posterior predictive exponentially weighted moving average control charts. As of yet, no validation has been conducted in the manufacturing sector. Riaz et al. [43] employed a lab-scale distillation column to combine the findings of numerous heterogeneous defect detection and identification approaches to overcome the limitations of individual heterogeneous defect detection and identification approaches. Ghosh et al. [44] merged the findings of various approaches for detecting and identifying defects in industrial processes using a fusion system. As a step in preprocessing data, resampling was used to improve the performance of the fusion system. To merge judgments from several models, the Dempster–Shafer evidence theory was applied. From categorization to fault assessment, Bayesian and machine learning approaches have been applied to several production phases and industrial processes.

In-process form and inspection data have never been merged for monitoring dimension product health, to the best of our knowledge. The most common approach to detecting the condition of finish-machined components, specifically surface metrology features, has been based on machine learning algorithms that monitor only the machining process and do not improve predictions when new data becomes available. An approach based on multisensor fusion is presented in this paper to address this deficiency. Data collected from various manufacturing phases will be used to develop an intelligent, dimensional product health monitoring system that delivers probabilistic predictions about the final product's status. A Bayesian information fusion technique is developed to update this forecast using fresh measurements, such as OMP. A Bayesian updating procedure combines machine learning knowledge with new information collected from OMP. This paper evaluates the performance of an EN24T steel-bearing housing component fabricated based on a case study. Manufacturing procedures include all stages of production, including heat treatment, grinding, hardness testing, machining, and in-process and postprocess inspections.

## 3. Methods and Materials

### 3.1. Data Fusion Background

Many applications of data fusion can be found, including surveillance and reconnaissance, environmental monitoring, and environmental danger identification [2–5]. It has been possible to integrate multiple sensors under heterogeneous data configurations using several approaches described in the literature. Multiple approaches to data fusion are being developed due to the numerous sensors and the heterogeneous nature of the data. Several fields were involved in developing these approaches, such as machine learning, pattern recognition, and statistical estimation. As a result of this extensive literature, traffic engineering has naturally benefited. A wide range of methodologies can be applied, depending on the application, ranging from sample arithmetic means to more advanced DF approaches. A three-way split might be proposed:

- Data mining engines use weighted combinations, multivariate statistical analyses, and their most modern versions to evaluate data. Using the arithmetic mean is the simplest way to merge data. Because estimators and classifiers perform differently, this method is not applicable [7–9].
- Multisensor data fusion typically relies on stochastic approaches, including Bayesian networks and state-space models [10], expectation-maximization methods and Kalman filter-based data flow (DF) [11, 12], possibility theory [13], probative reasoning, and especially proof theory [14–16]. A Bayesian approach may be considered an extension of this method [15–17].
- AI, genetic algorithms, and neural networks all belong to the domain of neural networks and artificial cognition. This latter method is often used both to create classifiers and estimation methods and to create fusion architectures between classifiers and estimators [6, 8].

Although DF approaches have been used to model complex systems for a long time, their use in transportation systems is gaining popularity [18–20]. DF approaches might be able to deliver the expected advantages in the case of road traffic. Although such techniques are feasible and effective, analyzing their feasibility, effectiveness, and utility presents considerable challenges [21–23]. With the introduction of ITS, DF has become an increasingly popular topic in the traffic engineering literature. The DF was first discussed in Sumner's article in the early 1990s [24]. In ITS systems, DF plays an essential role in enhancing efficiency. The use of DF in engineering has been discussed in several articles [22, 25, 13].

In order to better manage traffic on streets and highways, data processing methods designed by the Department of Defense can now be used [26–29, 45]. Data fusion in the DoD is organized hierarchically into five stages. Data from the source is processed at Level 0 as a preliminary step. Data can be normalized, formatted, sequenced, compressed, and batch processed [2, 28]. There may even be a method for identifying subobjects or data characteristics that will be used in Level 1 processing. Traffic management at the Level 1 level involves the collection of data from all relevant sources, including real-time point and wide-area traffic flow sensors, transit operators, toll data, cell phone calls, emergency call carton reports, investigating vehicle and roving tow truck texts, and commercial truck transmissions [13, 46]. Based on Level 1 processing, Level 2 processing identifies the likely scenario behind observed data and events using additional sources and databases. In addition to patrol reports and databases, road layout drawings, local and national weather reports, traffic mix predictions, and dates of construction and special events, this information can also be compiled using data from patrol reports and databases. The Level 3 processing identifies traffic patterns based on the likelihood of traffic events (e.g., traffic congestion, incidents, construction, preplanned special events, fires, or police actions) affecting traffic flows. During level 4 processing, predictions and evaluations are continually improved, and new sources of information are analyzed to enhance the overall data fusion procedure. There are times when a sixth level is added to address concerns regarding an individual's ability to comprehend and implement the conclusions reached by the fusion process. According to traffic literature, the DF process includes fundamental functions such as aligning input data chronologically or geographically, combining data, and mining data for knowledge extraction. It is also possible to achieve this goal through the fusion of multiple sources of information [30].

### 3.2. Neural Network

The neural network is based on biological nervous systems, which utilize many parallel features. A structure consists of an input layer, one or more hidden layers, and an output layer. Interconnected neurons receive related information during the preceding layers [47]. Data collected and analyzed by neural networks can recognize patterns, categorize data, and predict the future. Compared with a group data management technique and linear regression, Ghritlahre and Verma [48] concluded that neural networks produced the lowest error rate. As reported by Ghritlahre et al. [49], neural networks are superior to group data handling methods when it comes to predicting the thermal performance of a solar air heater. Ghritlahre and Prasad found that the neural network model had the lowest root mean square error compared to numerous predictive models. According to Ghritlahre and Prasad [50, 51], radial basis function networks provide the best energy efficiency for solar air heaters. With 14 neurons and one hidden layer, Ghritlahre and Prasad [52] could predict the performance of a solar air heater with a shallow error rate.

### 3.3. Machine Learning and Data Fusion for Industrial Forecasting

To classify and analyze industrial prognosis literature, a variety of criteria can be used, including the industrial sector, the data handled by the models, and the asset/process for which prognostic models are helpful. As opposed to prediction as a data-driven approach designed to attain one of three objectives, this research focuses on the following:

- Using the data collected in the industrial plant, characterize the investigated use case without making assumptions about its origin or significance. As a result, descriptive prognostic models do not depend on any a priori assumptions that might affect their insights, focusing instead on blind, unbiased extraction of added value. Many documented practical applications of industrial prediction rely on clustering algorithms and outlier detection methods.
- Predict when and how a failure in monitored equipment will occur and its consequences. Predictive prognostic models will typically use historical fault data from which a learning algorithm can determine whether a particular asset's data are associated with a particular target variable (such as a probability, severity, or the point in the process chain where the fault occurs). Supervised learning is predominant in machine learning.
- As soon as a plant malfunction alert is received, prescribe optimum actions. A prescriptive prognostic model adjusts the operational parameters and variables of the industrial process to reduce the likelihood of a fault occurring before a predictive model raises an alert. By optimizing rerouting assets or allocating human resources for unscheduled repairs, a model from this category would minimize the impact of a confirmed fault on an industry's output.
- Predictive prognostic models are often used to determine this scenario's objectives, often treated as an optimization problem. Optimization solutions dominate this category.

**Figure 1:**
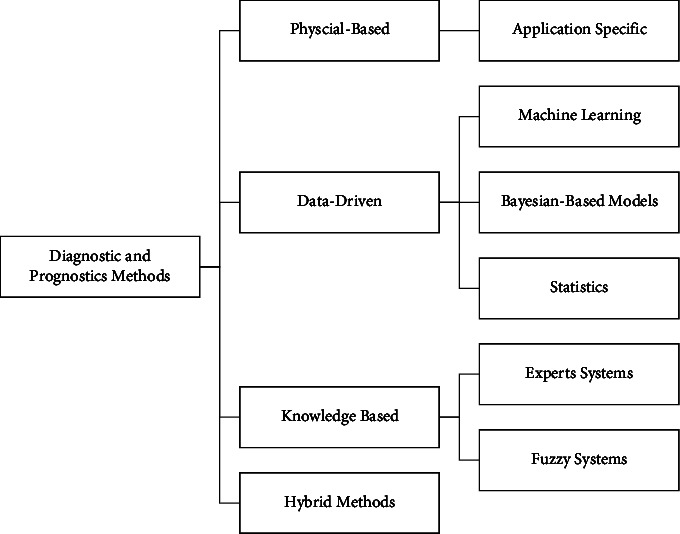
Industrial prognostics scenarios and data-driven methodologies.

Despite the categorization, contributions to the industrial prognosis literature will not be distinguished and categorized only as descriptive, predictive, or prescriptive. For multiple objectives, distinct model types are often hybridized to meet the needs of a particular application situation. In one of the most representative and intuitive examples of this combination of approaches, predictive prognosis—e.g., predicting whether a machine will have a fault—is combined with prescriptive prognosis—adjusting the machine configuration so that faults are less likely to occur. This analytical criterion will now analyze the most recent literature on industrial forecasting. Data-based prognosis has been studied in several industrial areas in recent years, including models and data fusion methods.  Figure 1 shows the industrial prognostics scenarios and data-driven methodologies discussed in this study.

### 3.4. Establishment of Production Cost Prediction Model

#### 3.4.1. Establishment and Quantification of Index System

Several factors influence manufacturing costs. In summary, production costs are composed of direct materials, direct labor, and manufacturing overhead. Predicting production costs is primarily guided by factors related to space and time. Due to the intricate impression aspects in various periods and the human factor inherent in historical data, we do not focus much on the time-influencing variables. Among the spatial elements of manufacturing costs, site circumstances and natural disasters play a significant role. To assess the impact of geographical factors on product production, the complexity coefficients are computed using the unconfirmed assessment model. Based on the current financial system, the policy elements are computed directly.

**Figure 2:**
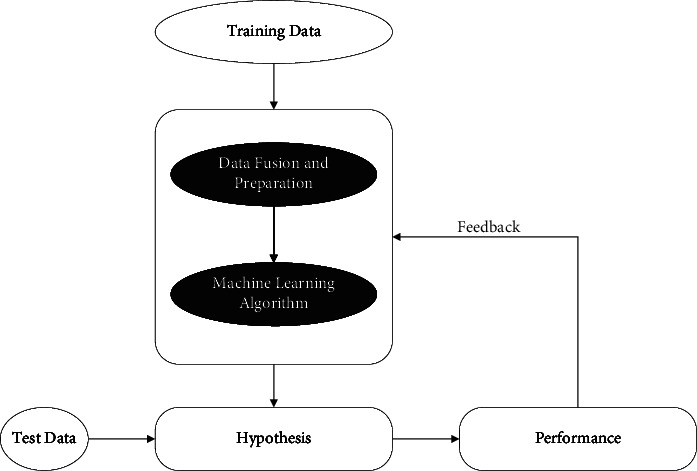
The basic structure of the proposed method.

In type 1, cost items are evaluated not only by space elements but also by time factors; in type 2, cost items concentrate mainly on time factors; and in type 3, cost items are determined by national and business financial policies. As a result, it becomes essential to accurately describe normalcy to identify deterioration patterns or trends, following the basic structure in Figure 2. Using mathematical algorithms (machine learning models) on training data obtained from the process or asset under investigation, one can describe behavioral patterns of interest. A variety of problems (hypotheses) can then be solved with new, unknown data (test data), including prediction, classification, and anomaly detection. Modern monitoring systems and intelligent devices require enormous amounts of data and extra information, making this task particularly challenging.

#### 3.4.2. Cost and Quality Relationship System and Impact

**Figure 3:**
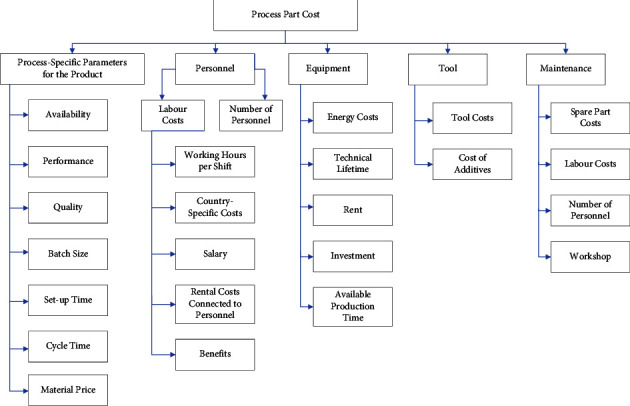
Cost factors and parameters affecting process parts.

In order to meet industry growth requirements and cost projections, manufacturing cost components will change from being uncontrollable to controllable as time passes and the firm expands.  Figure 3 shows the current relationship between production cost and quality in the manufacturing industry and its impacting factors.

**Figure 4:**
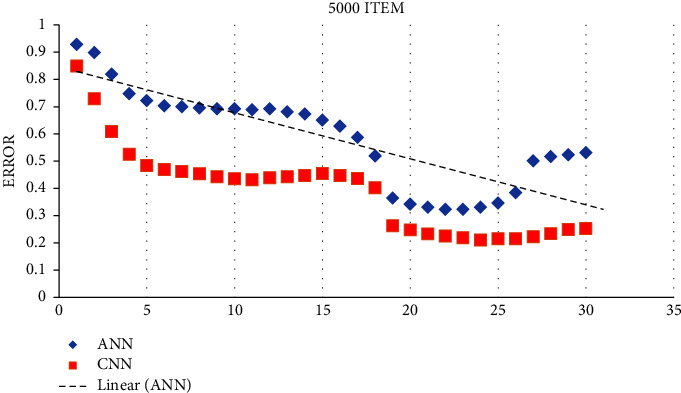
Prediction result obtained by applying the training and test prediction model (5000 items).

There is no guiding concept or guideline for choosing which several layers of layer nodes to use in the ANN. In order to obtain an adequate number of hidden layer nodes, it is necessary to repeat the process several times. The training error for neural networks with hidden layer nodes is calculated based on the exact training durations (3000 and 5000 times). Based on the training prediction model, Figure 4 shows the prediction result for the test sample.

## 4. Prediction of Production Costs Using a Multisource Information Fusion Machine Learning

**Figure 5:**
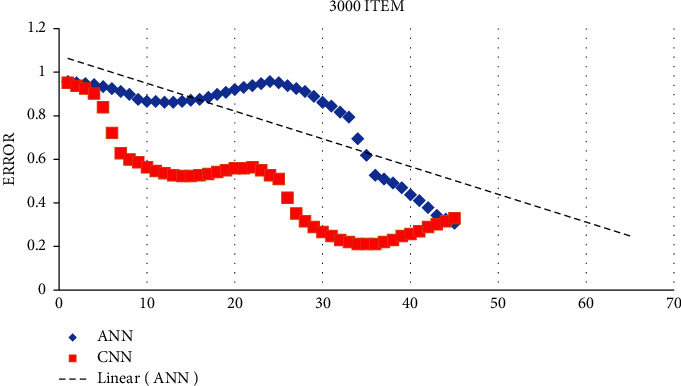
Prediction result obtained by applying the training and test prediction model (3000 items).

**Figure 6:**
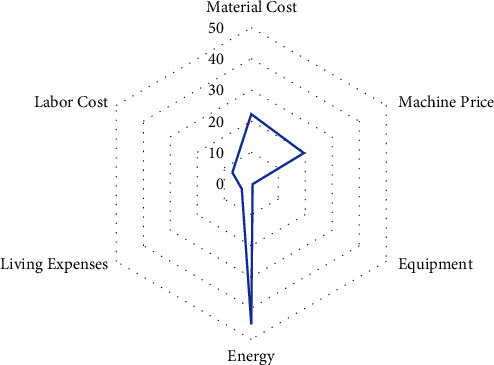
The true value of various costs in the production model.

The learning and performance of the ANN are affected by the number of layers of layer nodes. However, there are no guidelines or concepts for choosing them. Repeated attempts are needed to obtain enough hidden layer nodes. Training a neural network with various hidden layer nodes using the training sample set results in Figures 4 and 5. Figures 4 and 5 illustrate the training errors for the same training periods. When there are too few or too many nodes, the error gained from training the number of model nodes may be substantial. Figures 4 and 5 illustrate it. As the number of training sample sets rises, both ANN and CNN neural network models decrease their prediction errors, with CNN performing better than ANN. An analysis of the quantitative link between the various cost predictions and the actual value in production is conducted with a neural network with 50– 82 nodes. The impact of various cost categories on the real value of various expenses is shown in Table 1 of the production model. There is a need to compare the predictions of the neural network for each of the six major categories of production costs in order to determine which one is the best.  Table 2 shows that the CNN predicts the production cost better than the ANN. In contrast, Table 3 shows the overall outcomes of the production cost prediction compared to CNN (see Figure 6).

**Figure 7:**
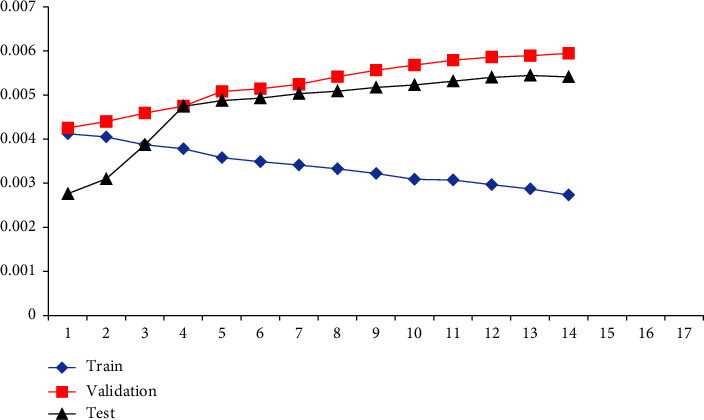
Performance value of the neural network.

**Figure 8:**
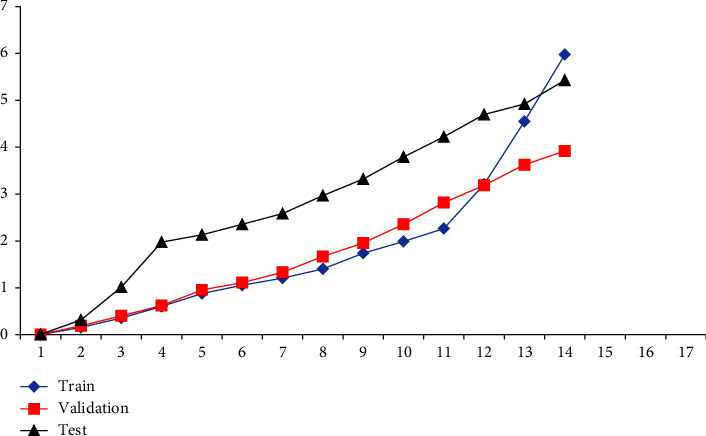
Performance value of CNN.

After the MATLAB neural network analysis, performance metrics are presented in Figures 7 and 8. The performance value graph trains, verifies, and evaluates input and output data to predict tool wear.

Figures 7 and 8 show that CNN is better at predicting the real value in each of the six cost categories than the ANN. In this case, CNN network has a prediction error of 0.0234 for the current month's total income. As a result of its superior prediction result and more effortless training procedure, CNN is better suited for combining multisource information than ANN. Furthermore, the two types of neural networks misestimate indirect costs, such as direct material costs and item consumption prices. ANN has a prediction error of 0.0453, and CNN has a prediction error of 0.0234 in the direct material cost prediction. Direct material costs are the sources of huge mistakes since they are unpredictable, including commodity prices and client purchases. These contribute to direct material costs fluctuating wildly. Several advantages of neural networks include their ability to train and customize, their resilience and fault tolerance, and their parallel structure and distributed storage capability that enables the rapid realization of nonlinear input-to-output mappings. Using a specific learning technique and a specific topological structure, neural networks can rapidly absorb samples' knowledge via offline learning. Once the connection weights and thresholds are saved, the trained neural network can quickly integrate the fusion of the system's input data and output the fusion results. A single sensor may not be able to accurately estimate targets due to the increasing complexity of modern technology.

## 5. Discussion

An integral part of the Industry 4.0 revolution is the concept of intelligent services and “servitization,” which reinvent asset maintenance. To minimize output damage, it is imperative to allow production line assets to fail early and predict why, how, and when they will fail, as well as to respond autonomously to these failures, including self-healing capabilities. In the new aftersales industry, factory equipment is maintained in a proactive, intelligent manner rather than a preventive or corrective manner. Creating a cloud-based service to deliver personalized and prognostic services would be possible by vertically integrating data monitored at the asset level with service processes in cloud-based back-end systems. These cloud-based technologies will reduce unscheduled equipment breakdowns and maintenance costs. As part of the integrated production and processes, the workbench components and the asset or product will have intelligence embedded. Thus, this decentralization will increase the importance of addressing emerging distributed computing paradigms, including edge analytics and privacy-aware federated learning, with profound implications for data fusion techniques and prognostic modeling. For data-based technologies to be implemented in business, highly trained and specialized personnel are required. Due to the manufacturing industry's digital transformation, data scientists, engineers, architects, database administrators, and business analysts are in greater demand. As a result, there is a lack of professional profiles that can fully leverage all asset information and manufacturing data in this area, a cause of difficulty in attracting and retaining bright professionals. Hopefully, more academic degrees in industrial forecasting will become available, and more staff training courses will be completed to resolve this problem. Intelligent services and “servitization,” reinventing asset maintenance, are another part of the Industry 4.0 revolution. It is essential to allow production line assets to fail early and predict why, how, and when, as well as to respond autonomously to this failure, including self-healing capabilities, to minimize output damage. In the new aftersales industry, factory equipment is maintained in a proactive, intelligent manner rather than a preventive or corrective manner.

Creating a cloud-based service to deliver personalized and prognostic services would be possible by vertically integrating data monitored at the asset level with service processes in cloud-based back-end systems. These cloud-based technologies will reduce unscheduled equipment breakdowns and maintenance costs. As part of the integrated production and processes, the workbench components and the asset or product will have intelligence embedded. Thus, this decentralization will increase the importance of addressing emerging distributed computing paradigms, including edge analytics and privacy-aware federated learning, with profound implications for data fusion techniques and prognostic modeling. Data-based technologies necessitate highly specialized and technical personnel to implement them in business. Due to the manufacturing industry's digital transformation, data scientists, engineers, architects, database administrators, and business analysts are in greater demand. As a result, there is a lack of professional profiles that can fully leverage all asset information and manufacturing data in this area, causing difficulty in attracting and retaining bright professionals. Hopefully, more academic degrees in industrial forecasting will become available and more staff training courses will be completed to resolve this problem. Businesses must predict product costs, which, in part, affect pricing, cost analysis, and management in terms of efficacy and scientific nature. Fusing data from multiple sources will undoubtedly become essential for controlling and processing sophisticated manufacturing equipment and warfare systems. A method for developing and evaluating the information fusion system and correctly evaluating its results are essential to achieving the results of multisource data fusion. Information fusion has just begun to develop and be applied as a relatively new field. Future developments in information fusion technology will follow this course. As needed, neural network modules can also be added to enhance tracking and prediction capabilities in actual applications.

## 6. Conclusions

In this article, we have covered several methodologies for data fusion and machine learning algorithms within the context of the Industry 4.0 paradigm. Depending on their primary objective, prognostic schemes can be classified as descriptive, predictive, or prescriptive. An assessment of the various methodologies available within each category has been conducted to conduct a well-informed analysis of the research activity in this field, focusing mainly on the challenges and industries that have implemented the reported approaches. The literature review identifies research trends and directions in data-driven industry forecasting that will capture the research community's attention. Due to the implementation of data-based modeling and fusion, there are some significant questions and open technical challenges, not only regarding highly imbalanced data, nonstationarity, and heterogeneity of information but also regarding their application in real-world industrial settings. As a result of new developments in data-driven prediction, such as those in this study, such challenges will undoubtedly be resolved in the coming years. The implementation of data-based technologies requires highly specialized and technical personnel. In response to the digital transformation of their industries, manufacturers need data scientists, engineers, architects, database administrators, and business analysts. Thus, there is a shortage of talent who can fully leverage asset information and manufacturing data, making it challenging to attract and retain bright professionals in this area. It is hoped that more academic degrees will be offered in industrial forecasting and that more staff training courses will be conducted to resolve this problem.

## Figures and Tables

**Table 1:** The true value of various costs in the production model.

Item costs	Actual cost (million dollars)
Material cost	22.24
Machine price	19.45
Equipment	0.45
Energy	45.21
Living expenses	3.5
Labor cost	7

**Table 2:** Estimated production costs for ANN and CNN.

	True value	Estimated value	
		ANN	CNN
Cost of production	56.056	57.33	56.13
Difference predicted	0	0.52	0.3
Estimation error	0	0.0453	0.0234

**Table 3:** ANN and CNN predictions of total production costs.

	Average accuracy	Highest accuracy	Time
ANN	0.8563	0.7645	967
CNN	0.9535	0.8746	775

## Data Availability

The data used to support the findings of this study are available from the corresponding author upon reasonable request.
